# Vibration Exposure and Transmissibility on Dentist’s Anatomy: A Study of Micro Motors and Air-Turbines

**DOI:** 10.3390/ijerph18084084

**Published:** 2021-04-13

**Authors:** Harish Kumar Banga, Pankaj Goel, Raman Kumar, Vikas Kumar, Parveen Kalra, Sehijpal Singh, Sunpreet Singh, Chander Prakash, Catalin Pruncu

**Affiliations:** 1 Mechanical Engineering Department, Guru Nanak Dev Engineering College, Ludhiana, Punjab 141006, India; drhkbanga@gmail.com (H.K.B.); sehijpalsingh@yahoo.in (S.S.); 2 Department of Business Management, Guru Nanak Institute of Management and Technology, Ludhiana, Punjab 141006, India; pankajgoel456@gmail.com; 3 Production and Industrial Engineering, Punjab Engineering College, Chandigarh 160012, India; myself1417@outlook.com (V.K.); parveenkalra@pec.ac.in (P.K.); 4 Mechanical Engineering, National University of Singapore, Kent Ridge 119007, Singapore; snprt.singh@gmail.com; 5 School of Mechanical Engineering, Lovely Professional University, Jalandhar-Delhi, G.T. Road, Phagwara, Punjab 144411, India; chander.mechengg@gmail.com; 6 Department of Mechanical Engineering, Imperial College London, Exhibition Road, London SW7 2AZ, UK; 7 Design, Manufacturing & Engineering Management, University of Strathclyde, Glasgow, Scotland G1 1XJ, UK

**Keywords:** air-turbine hand piece, grip force, micro motor hand piece, vibration exposure, vibration transmissibility, dentistry

## Abstract

The use of dental hand pieces endanger dentists to vibration exposure as they are subjected to very high amplitude and vibration frequency. This paper has envisaged a comparative analysis of vibration amplitudes and transmissibility during idling and drilling with micro motor (MM) and air-turbine (AT) hand pieces. The study aims to identify the mean difference in vibration amplitudes during idling, explore different grasp forces while drilling with irrigant injection by the dentist, and various vibration transmission of these hand pieces. The study utilized 22 separate frequency resonances on two new and eight used MMs and two new and eight used ATs of different brands by observing the investigator at 16 different dentist clinics. The study adopted a descriptive research design with non–probability sampling techniques for selecting dentists and hand pieces. Statistical methods like Levene Test of Homogeneity, Welch ANOVA, independent t-test, and Games–Howell test were utilized with SPSS version 22 and MS-Excel. The results reveal that vibration amplitudes and vibration transmissibility when measured at position 2 are higher than in another position 1. Vibrations during idling for used MMs are more than AT hand pieces, and the used MM (MUD) and used AT (AUA) hand pieces differ due to their obsolescence and over-usage. Vibration amplitudes increase every time with the tightening of grasping of the hand piece. Vibration amplitudes for each grasping style of MM hand piece differ from all other grasping styles of AT hand pieces. Routine exposure to consistent vibrations has ill physical, mental, and psychological effects on dentists. The used hand pieces more hazardous as compared to newer ones. The study suggests that these hand pieces must be replaced periodically, sufficient to break between two operations, especially after every hand piece usage. Hence, the present research work can be further extended by creating some control groups among dentists and then studying the vibration amplitude exposure of various dental hand pieces and subsequent transmissibility to their body parts.

## 1. Introduction

The use of dental hand pieces endangers dentists to vibration exposure [1]. Hand-arm vibrations exposure measurements are specified, and limits are also defined in ISO 5349-1:2001. Dental personnel are subjected to the very high amplitude and frequency of vibrations. A recent study found that the dentist’s fingers’ sensation values were higher among ten female dentists having at least ten years of experience in the said field than among the selected personnel [2]. Tingling and numbness in the fingers and episodic blanching of the fingers suggest Raynaud’s phenomenon when exposed to cold. The occurrence of Raynaud’s phenomenon in association with a history of exposure to vibrating tools and the absence of an underlying disease is termed hand-arm vibration syndrome (HAVS), formerly known as vibration white finger. Until recently, most research into this condition’s underlying pathophysiology has focused on the circulatory disturbances as the primary mechanism. It is now appreciated that the vascular changes and the neurological changes are likely to occur independently of each other [3].

ISO 5349:2001 describes the hand pieces vibration exposure-response mean acceleration amplitude in the 1/3rd octave band of 6.3–1250 Hz. The risk prediction model contains many uncertainties, such as a change in individual susceptibility and other related factors, a change in work methods, and uncertainty in predicting total effective vibration exposure. The consequence of aging on the vibration amplitude of dental hand pieces is not entirely known [4]. Hand-arm vibration amplitudes above 2.5 m/s^2^ are harmful [5,6]. There is significantly less information available on the physical and other health issues related to dental hand pieces. Dental personnel who are subject to high vibration magnitude in the dental environment have a risk of developing neurological and other circulatory disturbances in their hands. The risk of injury to women dentists in Sweden was higher than among other dental personnel [7].

A study that depicts the relationship between vibration amplitude and musculoskeletal disorder (MSD) symptoms in dental hygiene students showed that MSD symptoms might increase abruptly using manual and ultrasonic hand pieces [8]. The effect of grip force, hand posture, and the accelerometer’s location on hand piece on vibration amplitude were also studied [9]. Through vibration exposure from a specially designed handle, researchers looked at gender differences in the amount of vibration absorption per unit of time. The male participants had substantially higher vibration absorption than females [10]. The shoulder, neck, and arm muscle were placed under the most stress by routine dental work in an electromyographic study. The discomfort may vary from mild to severe or incapacitating at times.

In most cases, the early symptoms of musculoskeletal disorder (MSDs) among these workers were mild to unbearable pain, swelling in body parts, tenderness, impassiveness, stinging feeling, and sometimes partial to complete loss of essential strength [11]. The studies have also revealed that repetitive and vigorous labor can lead to tendinopathies and carpal tunnel. Most of the complex operations are handled by dental workers’ hands, leading to moving ligaments to tendon sheaths with synovial fluid. Consequently, repetitive and vigorous labor with vibrating tools causes fluid accumulation, soreness, and irritation [12].

The present research work has attempted to contribute to the existing resources upon evidence on ergonomics, creating general awareness of common ergonomic vulnerability factors for dental practitioners. It has also been endeavored to offer specific alternatives to dental practitioners’ attention regarding professional circumstances and work practices. The main aim is to provide a comfortable and productive scenario for these practitioners [13]. A few research works confirm that dental workers often deal with posture issues and limb pain. The pain is mainly observed in their shoulders, back, arm, hand, neck, and wrist due to poor seating postures, improper positioning of their clients, and inadequate working methods. This pain also covers elbow, hand, and wrist flexion leading to severe thumb hyperextension, stressful neurovascular structures and, ligaments [14]. The foremost dental tissues eliminated in simple restorative approaches are tin and dentine. The underlying pulp is concerned with the reactionary responses per disease rather than trauma [15]. In a damaging oral environment, the sickness procedure concerning caries gradually destroys these tissues. Once effective intervention becomes necessary, restoration typically involves reducing instruments’ usage within arrangements for placement on a filling material [16]. Various equipment was used because of the growth access to caries for the removal of diseased tissue, and the use of rotary hand pieces and their associated instruments remain common. The development of these tools underwent a significant change into the 1950s; now, excessive speeds in these hand pieces are common [17]. There were persistent improvements in dental gadgets over the past years. However, there are possible implications for dental workers’ long-term health in terms of auditory damage along with issues in the upper limbs. The tools can also be broken through repeated use [18]. Understanding this equipment’s physical characteristics can help us to identify the issues they may cause and conduct design upgrades. To date, there is still a paucity of studies on these hand pieces [19]. However, present-day strategies such as logging biometric data and accelerometer attachments present an opportunity for assessing the vibrations of dental hand pieces [20,21,22].

High-speed drills and ultrasonic scalers expose the hands of the users to high-frequency vibration. The symptoms of vibration injuries were studied through a questionnaire of 374 Swedish female dental personnel who had reported damage due to the excessive hand-arm vibrations. They were asked about the neurological and vascular symptoms in their thumb, fingers and hands, decreased hand strength, tremor, and pain in their elbow, hands, fingers and neck. The study evaluated the overall vibration exposure among these dental personnel through their yearly self-reports and daily exposure to hand-arm vibration. The vibration levels of the tools used were assessed by examining former studies. The most common symptoms were numbness and loss of strength. Pain in the hands and symptoms from the neck/shoulders were also frequent complaints. In most cases, the dental professionals’ neurological symptoms in their hands and fingers were more recurrent than other vascular symptoms. Before the first appearance of symptoms, hand-arm vibration exposure had a mean period of almost 6.8 years. The daily duration of vibrations was measured and quantified in ten dental professionals at their exposure during the ultrasonic scalers’ usage. This daily exposure ranged from 0 to 50 min, with an average of 12 min. The self-assessed duration of exposure was overestimated [23]. All tasks require workers to use their muscles to exert some level of force; however, when a job requires them to exert a particular muscle too strenuously, it can damage the muscle, related tendons or joints, and/or other soft tissue [24].

Furthermore, dental practitioners were observed to statically hold postures requiring more than 50% of the body’s musculature to contract. This results in increased muscular effort, leading to muscle overload, decreased blood flow, and increased pressure on muscles and joints [25,26]. The risk of developing an MSD increases when similar body parts are used continuously, with few breaks or chances for rest. Highly repetitive tasks can lead to fatigue, tissue damage, discomfort, and, eventually, injury. This can occur even if the level of force is low and the work postures are not awkward [27,28]. Dental hygienists commonly report work-related musculoskeletal disorders, and the significant causative factors are repetitive motion, pinch grasp, force vibration and prolonged awkward working positions [29]. The vibration transmissibility investigated forearm muscle activity and subjective grip dexterity using various gel-based compositions and designs [30]. The hand-arm vibration, noise exposure, and shift in hearing threshold were assessed for the prolonged use of handheld tools used in three different occupations [31].

The reviewed literature reveals that dental hand pieces endanger the dental personnel to vibration exposure, and the vibration of dental hand pieces may be harmful in the long run. Dental personnel are subjected to very high amplitude and frequency of vibrations. There is significantly less information available on the physical and other health issues related to dental hand pieces. Personnel subject to high vibration magnitude in the dental field face a risk of developing neurological and other circulatory disturbances in their hands. This work aimed to measure the vibration amplitude of new and used hand pieces and analyze these hand pieces’ frequencies. Vibration amplitude and the transmissibility of dental hand pieces were measured during normal clinical work using different headpieces based on their time of use. Work was also simulated by using irrigant injection at the maximum and minimum levels. The effect of grip force and grasping position on vibration exposure of headpieces was also studied.

In the present research work, a novel idling assisted drilling operation was proposed. The experimented biometric setup included an accelerometer, facts logger, and an evaluation software to study the influence of different weighted vibrations as per ISO 5349:2001-1. Under the idling condition, the weighted vibrations in old micro-motor hand pieces were higher than those in newer hand pieces.

This paper is organized as follows. Section 2 includes information on handheld tools used in the experiment and covers the research methodology, research objective, and hypotheses. Section 3 deals with the analyses of the results and provides a discussion on the vibration amplitude during idling, analysis of drilling with different grip forces, micro motor and air-turbine, analysis of various statistical techniques, and analysis of the vibration transmissibility of micro motor and air-turbine. Section 4 includes the major conclusions followed by Section 5 which contains policy implications, suggestions, and the future scope of the study.

## 2. Materials and Methods

### 2.1. Hand Held Tool

The weighted vibration for declaration purposes is measured according to appropriate test standards. Tools are tested under conditions as appropriate for the type of tool and specified in the relevant Part 2 of the test standard. The requirements are given in Part 2 either supplement or modify the requirements given in Part 1 to account for the particular hazards and characteristics of these specific tools. The measurements method of the vibration described in these standards is based on the standard ISO 5349-1.

### 2.2. Handheld Dentistry Tools under Present Experimentation

This study mainly used air-turbine and micro-motors which are commonly used in the dentistry drilling process. The air-turbine is one of the famous abstracter devices used for cutting and drilling a tooth under operation. This device is characterized as lightweight, small in size and high-speed rotating device with less compressed air usage resulting in painless grinding. Its revolutions even go above 0.18 million rpm. On the contrary, a micro motor is characterized as a short, lightweight, slow or medium-speed electric motor device with high torque, resulting in better performance. Its revolutions are in the range of 35,000–36,000 rpm. These motors add to the power of the hand pieces and help provide a cutting speed over 0.18 million rpm.

### 2.3. Experimental Approach

In this study, 20 new and used micro-motor and air-turbine hand pieces were tested in the actual dental work environment at Dental Hospital in the University Institute of Dental Sciences, Panjab University, Chandigarh, India. The number and types of hand pieces are shown in Table 1.

**Figure 1:**
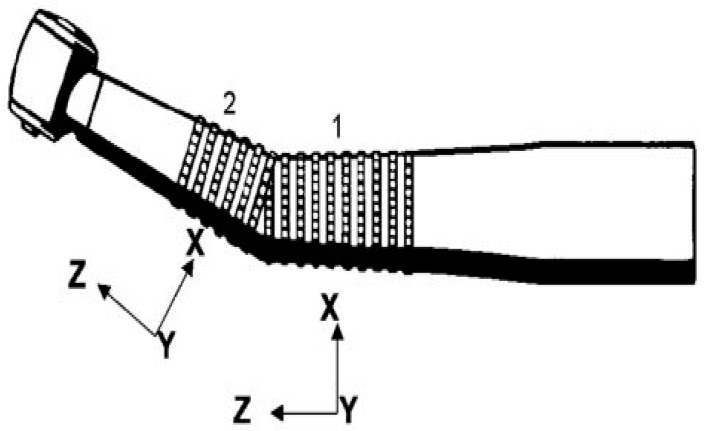
Location of measurement position and directions on air-turbine hand piece [32].

**Figure 2:**
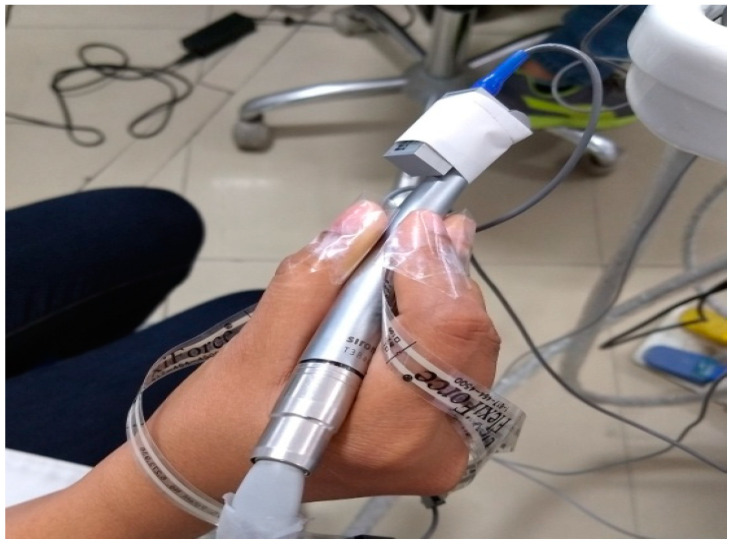
Accelerometer attachment.

In Table 1, 1 and 2 were new and 5–12 were used micro-motor hand pieces. Also, 3 and 4 were new, and 13–20 were used air-turbine hand pieces. The micro-motor hand piece rotational speed was 25,000–40,000 rpm, and the rotational speed of the air-turbine hand piece was 35,000–400,000 rpm. The speed of both hand pieces was restricted to 35,000 rpm. During testing, burrs made of steel with tungsten carbide coating were used. The vibrations were measured in all three directions. Two positions of the accelerometer attachment on the hand piece was selected based on previous studies. It was also confirmed by observations of the gripping of the hand pieces by various dentists. These positions are shown in Figure 1 [32]. Position 1 is the grasp position for light operations such as scaling, whereas position 2 is the grasp position for operations like drilling, which require a large amount of force. The maximum force position was chosen for gripping the hand pieces, and the tri-axial accelerometer was also attached at this location. The gripping and accelerometer attachment provided are shown in Figure 2.

The elbow-arm angle was fixed at 120°, and the wrist-hand angle was measured using a goniometer made by Physiopedia in London, United Kingdom. The elbow-arm angle was set at 120° in line with an optimized range of 110–130°, as reported in the literature. Consequences of the dentist’s gripping effort on the vibration amplitude at various hand locations were studied by applying varying forces. The forces were fixed at varying amounts of the dentist’s grip efforts: mild grip, gentle grip, moderate grip, and tight grip. These levels were fixed as per the subjective estimates of the dentist. Also, the effects of irrigant injection on the vibration of air-turbine hand pieces were tested. Air-turbine hand pieces used an inbuilt irrigant injection system.

**Figure 3:**
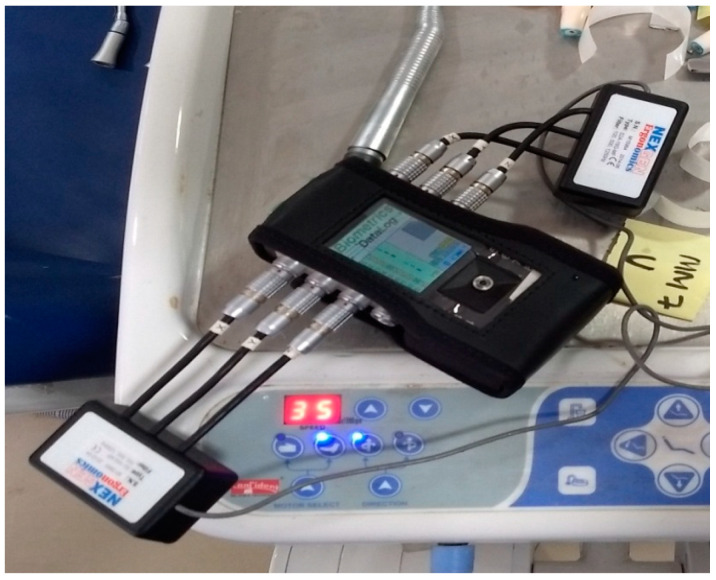
Data-logger with an accelerometer.

The impact of irrigant injection was studied only for air-turbine hand pieces. All tests were performed during idling. The RMS vibrations were analyzed in the 1/3rd-octave band in the frequency range of 6.3–1250 Hz. The total number of hand pieces tested was 20 during idling. The weighted vibrations were also calculated, and exposure checked specified in ISO 5349:2001-1. Drilling on actual teeth mounted on a manikin with all new and four used hand pieces was carried out. During the drilling of teeth, the irrigant injection was adjusted to a maximum and minimum value, and its effect was studied. The idling results depict that vibration amplitude obtained in position 2 sufficiently represents the vibration of hand pieces, and thus the subsequent tests were performed in this position. The measuring instrument was a Biometric setup, including the accelerometer (10 G, 16 G), the conditional amplifier (data logger), and the analysis software was manufactured by Smart Biometric Solutions, Texas Instruments, Texas, USA, as shown in Figure 3. The accelerometer was attached with glue and surgical tape to the proper measuring position of the hand piece.

The recorded readings were analyzed in VATS software by NexGen Ergonomics Inc., Quebec, QC, Canada, and corresponding weighted acceleration in a1/3rd-octave band in the frequency range 6.3–1250 Hz was calculated. The weighted vibrations were calculated, and exposure checked to limits specified ISO 5349:2001-1. The measured time of each dental hand piece represents the drilling time of the dentist on each patient.

### 2.4. Hand Piece Examination Research Methodology

The study undertook 22 separate frequency resonances (in Hz) on the fixed and restricted rotational speed (rpm) of two new and eight used micro motors and two new and eight used air-turbine hand pieces. These hand pieces belong to different brands of various national and international manufacturers. The investigator collected the frequencies of 20 hand pieces through personal observation at 16 different dentist offices in India. The study adopted a descriptive research design with non–probability sampling technique for the selection of dentists and hand pieces. The convenience sampling method was used in hand piece selection, and the Snowball sampling technique was been adopted the in case of a dentist visit. These dentists were visited by booking telephone appointments at their convenience. The sampling unit consisted of Micro Motors and Air-turbine hand pieces. Frequencies collection technique was the primary and first-hand information collection method. The frequencies of vibration amplitudes were observed, compiled, and evaluated with various statistical methods. Descriptive statistics, Levene Test of Homogeneity [33], independent t-test, Welch ANOVA [34,35], and Games–Howell test [36] were utilized and assessed with SPSS version 22 and MS-Excel. SPSS version 22 software is a statistical package developed by SPSS Inc., Chicago, IL, USA, and later acquired in 2009 by IBM as IBM SPSS Collaborations and deployment Services. MS Excel is a spreadsheet developed by the Microsoft Corporation, Washington, WA, USA.

### 2.5. Purpose of the Study

In analyzing the efficacy of the handheld tools, the following objectives were considered:

- To study the vibration amplitudes during Idling of micro motor and air-turbine hand pieces.
- To explore different grasp forces while drilling with irrigant injection by the dentists using different hand pieces.
- To study various vibration transmission of these hand pieces.

### 2.6. Null Hypotheses of the Study

To achieve the objectives mentioned above of the investigation, the study laid down the following hypotheses:

H_A1_: There exists a statistically significant difference in the mean vibration amplitudes of micro motor and air-turbine hand pieces during idling.

H_A2_: There is a statistically significant difference in grasp forces during drilling with the dentist’s irrigant injection for different hand pieces.

H_A3_: There exists a statistically significant difference in the vibration transmission of the hand pieces.

## 3. Result Analysis and Discussion

### 3.1. Vibration Amplitudes during Idling

Idling was done by fixing the dentist’s wrist angle at 175–185° with the required and fixed elbow angle of 120°. The time of the measurement was 20–25 s for individual vibration amplitude. Idling was performed for both micro motor (MM) and air-turbine (AT) hand pieces. The study was conducted using two new MMs, four used MMs, two new ATs, and four used ATs. Hence, a total of 12 hand pieces measuring 1/3rd–octave band vibrations amplitudes were studies. These machines were held at two positions, viz. position 1 and position 2. These hand pieces were coded, and their descriptive statistics were calculated and are shown in Table 2.

The study first tested the homogeneity of variance with the help of the Levene test [33]. It was expected that all hand pieces would have equal population variances in the independent observations of 1/3rd–octave band vibrations amplitudes [37] of both hand pieces. The Levene test showed that the computed variances for 1/3rd–octave band vibrations amplitudes for both hand pieces are not equal with F (11, 252) = 54.017, *p* = 0.000, and α = 0.05. Hence, the null hypothesis assuming equal variances in 1/3rd–octave band vibrations amplitudes was not accepted and rejected the feasibility of performing one-way ANOVA on the observations. ANOVA works upon an assumption of the equality of variances in population means. Hence, it is advisable to utilize a robust test of equality of means, i.e., Welch test for ANOVA [34,35]. This test did not assume the assumption of equal variances of means. The Welch ANOVA output showed a significant difference in means and variance between the means are not equal. F (11, 98.842) = 5.938, *p* = 0.000 and α = 0.05. It is important to know in which hand piece the mean 1/3rd–octave band vibrations amplitudes differ. A post-hoc comparison Games–Howell test was conducted to find the hand pieces with differing vibration amplitudes. Games–Howell is a suitable post hoc measure ignoring the differences in variance in the population mean of samples [38]. The study did not find any statistically significant differences in hand pieces’ mean distribution score except MUD and AUA. The output of the different hand pieces is shown in Table 3.

The study found that the mean vibration amplitude in case of MUD hand piece was different from MNA (*p* = 0.006, α = 0.05), CI (0.126 to 1.086), MNB (*p* = 0.004, α = 0.05), CI (0.147 to 1.106), MUA (*p* = 0.003, α = 0.05), CI (0.161 to 1.119), MUB (*p* = 0.002, α = 0.05), CI (0.183 to 1.140), MUC (*p* = 0.003, α = 0.05), CI (0.178 to 1.135), ANA (*p* = 0.003, α = 0.05), CI (0.164 to 1.123), ANB (*p* = 0.002, α = 0.05), CI (0.184 to 1.141), AUB (*p* = 0.004, α = 0.05), CI (0.148 to 1.108), AUC (*p* = 0.003, α = 0.05), CI (0.176 to 1.133) and AUD (*p* = 0.003, α = 0.05), CI (0.164 to 1.121). The study found that the mean vibration amplitude in case of AUA hand piece was different from MNA (*p* = 0.278, α = 0.05), CI (0.020 to 0.536), MNB (*p* = 0.014, α = 0.05), CI (0.042 to 0.556), MUA (*p* = 0.009, α = 0.05), CI (0.056 to 0.568), MUB (*p* = 0.004, α = 0.05), CI (0.079 to 0.589), MUC (*p* = 0.005, α = 0.05), CI (0.073 to 0.584), ANA (*p* = 0.008, α = 0.05), CI (0.059 to 0.572), ANB (*p* = 0.004, α = 0.05), CI (0.795 to 0.590), AUB (*p* = 0.013, α = 0.05), CI (0.042 to 0.559), AUC (*p* = 0.005, α = 0.05), CI (0.071 to 0.583) and AUD (*p* = 0.008, α = 0.05), CI (0.059 to 0.570).

The difference in the micro motor used hand piece (MUD) and used air-turbine hand piece (AUA) was due to overuse of both hand pieces. The MUD was in use for more than two and a half years. The AUA was in use for more than three years, impacting the dental professionals’ overall professional idling efficiencies. Therefore, the idling efficiency of both used hand pieces were different from the other hand pieces under study.

### 3.2. Drilling with Different Grip Force: Micro Motor and Air-Turbine

The study proceeded with an evaluation of drilling with different grip or grasp forces by the dentists. There are four types of grip/grasp forces, viz. mild grasp, gentle grasp, moderate grasp, and tight grasp. This drilling was performed with micro motors (MM) and air-turbine (AT) hand pieces. The study was conducted upon one MM and one AT at 22 differing frequencies of these hand pieces. Hence, there is a study of overall two hand pieces measuring 1/3rd–octave band vibrations amplitudes during drilling with irrigant injection at position two for the dentist’s varying grasp forces. These machines are held at two positions, viz. position 1 and position 2. Table 4 shows various 1/3rd-octave band vibration amplitude of Micro motor and air-turbine hand piece under four grasping conditions. It was expected there would be a difference in the vibration amplitude at the different frequencies of these hand pieces. An independent t-test was run to evaluate the difference in the mean distribution of these hand pieces respective grasp positions. The descriptive output is summarized in Table 5.

This test is essential to fulfil the assumption of homogeneity of variances and can be tested with the Levene test of homogeneity. This test verifies that the distribution of the cores between the variables assumes equal variance or not. In the present grasping conditions, this test verified this assumption at *p*-value > 0.05. The output derived is summed up in Table 6.

The study showed that all grasping conditions testing homogeneity of variances in two hand pieces, *p*-value < 0.05 depicting equal variances, have not been assumed. An independent t-test was run with the present assumption of not equal variances assumed. An independent t-test was performed between various greasing conditions of micro motor and air-turbine at 1/3 –octave band vibration amplitude at 22 different frequencies. The study showed that the output was statistically significant in mild grasping conditions, t (23.330) = 4.202, *p* = 0.000, α = 0.05, thus rejecting the null hypothesis that there exists a statistically significant difference between computed scores of MMMG with (M = 0.039, SD = 0.352, *n* = 22) and ATMG with (M = 0.0066, SD = 0.0083, *n* = 22) with CI 0.016 ± 0.048. Hence, mild grasping conditions for both hand pieces differ. In another test for gentle grasping conditions, the study showed that output is statistically significant in gentle grasping conditions, t (22.751) = 4.620, *p* = 0.000, and α = 0.05. The null hypothesis was rejected that there exists a statistically significant difference between computed scores of MMGG with (M = 0.0458, SD = 0.390, *n* = 22) and ATGG with (M = 0.0066, SD = 0.0080, *n* = 22) with CI 0.022 ± 0.057. Hence, gentle grasping conditions for both hand pieces also differ. The third investigation was made for a moderate grasping condition for both hand pieces by the dentists. The study showed that output is statistically significant in moderate grasping conditions, t (22.650) = 4.424, *p* = 0.000, and α = 0.05. The null hypothesis was rejected that there exists a statistically significant difference between computed scores of MMMoG with (M = 0.0457, SD = 0.0404, *n* = 22) and ATMoG with (M = 0.0069, SD = 0.0080, *n* = 22) with CI 0.021 ± 0.057. Hence, moderate grasping conditions for both hand pieces also differ. The last investigation was made for a tight grasping condition for both hand pieces by the dentists. The study showed that the output is statistically significant in tight grasping conditions, t (25.622) = 3.881, *p* = 0.000, α = 0.05. Null hypothesis was rejected that there exists a statistically significant difference between computed scores of MMTG with (M = 0.0502, SD = 0.0436, *n* = 22) and ATTG with (M = 0.0122, SD = 0.0146, *n* = 22) with CI 0.018 ± 0.058. Hence, tight grasping conditions for both hand pieces also differed and the mean vibration amplitude for both hand pieces differ for all grasping conditions. There may exist differences in the overall mean vibration amplitude of these hand pieces grasping conditions when studied collectively. Hence one-way ANOVA may be the suitable option in this case. The descriptive statistics of differing grasping conditions are shown in Table 7.

The study first tested the homogeneity of variance in vibration amplitudes of various grasping conditions with the Levene test’s help. It was expected that all hand pieces would have equal population variances in the independent observations of 1/3rd–octave band vibrations amplitudes of both hand pieces. The Levene test showed that computed variances for 1/3rd–octave band vibrations amplitudes for various hand pieces various grasping conditions are not equal with F (7, 168) = 20.922, *p* = 0.000, and α = 0.05. Hence, the null hypothesis assuming equal variances in 1/3rd–octave band vibrations amplitudes of various grasping conditions was not accepted and rejected the feasibility of performing one-way ANOVA on the observations. ANOVA works upon the assumption of the equality of variances in the population means. Hence, it is advisable to utilize a robust test of equality of means, such as the Welch test for ANOVA. This test did not assume the assumption of equal variances of means. The Welch ANOVA output showed that there was a significant difference in means, and variances between the means were not equal. F (7, 70.486) = 10.788, *p* = 0.000 and α = 0.05. It is important to know how grasping condition with specific mean 1/3rd–octave band vibrations amplitudes differ. A post-hoc comparison Games–Howell test was conducted to find hand piece with differing grasping conditions. Games–Howell is a suitable post hoc measure ignoring the difference in variance in a population mean of samples.

The study did not find any statistically significant differences in the intra hand piece mean distribution score under various grasping conditions. However, there exist significant statistical differences in inter-hand piece mean distribution score under different grasping conditions. The output of the differing grasping conditions is shown in Table 8.

The study found that the mean vibration amplitude of mild grasping condition (MMMG) did not differ statistically from other micro-motor conditions. However, the mild grasping condition of micro motor is different from the mild grasping condition of air-turbine (ATMG) with (*p* = 0.007, α = 0.05), CI (0.007 to 0.058),gentle grasping condition ATGG with (*p* = 0.007, α = 0.05), CI (0.007 to 0.058), moderate grasping condition ATMoG with (*p* = 0.007, α = 0.05), CI (0.007 to 0.058) and tight grasping condition ATTG with (*p* = 0.046, α = 0.05), CI (0.000 to 0.053). The mean vibration amplitude of the gentle grasping condition (MMGG) did not differ statistically from other micro-motor conditions. However, the gentle grasping condition of micro motor was different from mild grasping condition of air-turbine (ATMG) with (*p* = 0.003, α = 0.05), CI (0.011 to 0.067), gentle grasping condition ATGG with (*p* = 0.003, α = 0.05), CI (0.011 to 0.067), moderate grasping condition ATMoG with (*p* = 0.003, α = 0.05), CI (0.011 to 0.067) and tight grasping condition ATTG with (*p* = 0.015, α = 0.05), CI (0.005 to 0.063). The mean vibration amplitude of the moderate grasping condition (MMMoG) did not differ statistically from other micro-motor conditions. However, the moderate grasping condition of micro motor is different from mild grasping condition of air-turbine (ATMG) with (*p* = 0.004, α = 0.05), CI (0.010 to 0.068), gentle grasping condition ATGG with (*p* = 0.004, α = 0.05), CI (0.010 to 0.068), moderate grasping condition ATMoG with (*p* = 0.004, α = 0.05), CI (0.010 to 0.068) and tight grasping condition ATTG with (*p* = 0.021, α = 0.05), CI (0.003 to 0.064). The mean vibration amplitude of the tight grasping condition (MMTG) did not differ statistically from other micro-motor conditions. However, the tight grasping condition of micro motor was different from mild grasping condition of air-turbine (ATMG) with (*p* = 0.003, α = 0.05), CI (0.012 to 0.075), gentle grasping condition ATGG with (*p* = 0.003, α = 0.05), CI (0.012 to 0.075), moderate grasping condition ATMoG with (*p* = 0.003, α = 0.05), CI (0.012 to 0.075) and tight grasping condition ATTG with (*p* = 0.013, α = 0.05), CI (0.006 to 0.070). As per the study’s prior expectations, the difference was that MM and AT’s grasping style in dental professionals differs. Hence, the study showed a clear-cut difference in dentists’ particular grasping styles in MM and AT, respectively. These electrically driven micro motors have an edge over the air-turbine while grasping and drilling in dental operations.

### 3.3. Vibration Transmissibility: Micro Motor and Air-Turbine

The study evaluated vibration transmissibility [39,40] during drilling with irrigant injections by the dentists. Basically, a dentist initiates with a tool point and then grasps the point and finally the wrist point. The frequencies were measured at three stages, viz. tool point to grasp-point, grasp-point to wrist end, and tool point to wrist end. These vibration amplitudes and transmission were measured for micro motors and air-turbine hand pieces. This drilling with irrigant injections was performed with four MMs and four ATs hand pieces. The selected hand pieces consisted of two new and two old micro motors and air-turbines, respectively. The study was conducted on 22 differing frequencies of the hand pieces, hence, eight hand pieces measuring 1/3rd–octave band vibrations transmissions during drilling with irrigant injection at position two for varying transmission points by the dentists were studies. These hand pieces were held at two positions, viz. position 1 and position 2. Table 9 shows various 1/3rd-octave band average vibration transmissions of MM and AT hand pieces under three transmission points.

**Figure 4:**
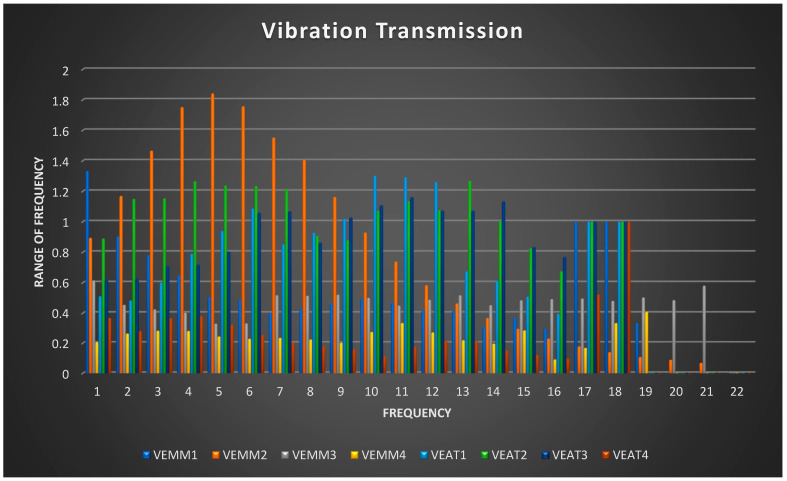
Showing 1/3rd-octave band average vibration transmission.

The 1/3rd–octave band average vibration transmissions shown in Table 9 on various 22 frequencies are shown graphically in Figure 4.

The resonance frequency range of vibration transmissibility for both used and new hand pieces (for both MMs and ATs) in a majority of the cases is less than unity. Hence, it shows that most dental professionals’ vibrations were absorbed through their fingers, palms, and hands. The lower the transmissibility range, the higher the absorption by the limbs of the dental workers. The statistical testing technique consisted of each hand piece average transmission with three transmission points for all the 22 frequencies. The descriptive statistics of average vibration transmissions were calculated and are shown in Table 10.

The study first tested the homogeneity of variance in 1/3 octave band average transmissions of various hand pieces with the help of the Levene test. It was expected that all hand pieces would have equal population variances in their independent observations of 1/3rd–octave band average vibrations transmissions. The Levene test showed that computed variances for 1/3rd–octave band vibrations amplitudes for various grasping conditions of both hand pieces are not equal with F (7, 168) = 12.673, *p* = 0.000, and α = 0.05. Hence, the null hypothesis assuming equal variances in 1/3rd–octave band average vibrations transmissions was not accepted and rejected the feasibility of performing one-way ANOVA on the observations. ANOVA works upon the assumption of the equality of variances in population means. Hence, it is advisable to utilize a robust test of equality of means, such as the Welch test for ANOVA. This test did not assume the assumption of equal variances of means. The Welch ANOVA output showed that there was a significant difference in means, and variances between the means were not equal. F (7, 69.933) = 18.531, *p* = 0.000 and α = 0.05.

It is important to know in which grasping condition with specific mean 1/3rd–octave band average vibrations transmissions differ. A post-hoc comparison Games–Howell test [36] was conducted to find hand piece with differing vibration transmissions. Games–Howell is a suitable post hoc measure also ignoring the difference in variance in population mean of samples. The study found statistically significant differences in both inter and intra hand piece mean distribution score of vibration transmissions. The output of the differing grasping conditions is shown in Table 11. The study found that the mean vibration transmissions did not differ primarily in statistical terms in inter-machine transmission mechanisms. The study found that the mean vibration transmissions of the new micro motor hand piece (VEMM1) were statistically different from the used micro motor hand piece (VEMM4) with (*p* = 0.015, α = 0.05), CI (0.039 to 0.534). The mean vibration transmissions of another new micro motor hand piece (VEMM2) were statistically different from used micro motor hand piece (VEMM4) with (*p* = 0.009, α = 0.05), CI (0.107 to 1.024) and used air-turbine hand piece (VEAT4) with (*p* = 0.015, α = 0.05), CI (0.077 to 1.020). The mean vibration transmissions of the used micro motor hand piece (VEMM3) was statistically different from used micro motor hand piece (VEMM4) with (*p* = 0.000, α = 0.05), CI (0.130 to 0.351), new air-turbine hand piece (VEAT2) with (*p* = 0.007, α = 0.05), CI (−0.730 to −0.082), used air-turbine hand piece (VEAT3) with (*p* = 0.043, α = 0.05), CI (−0.595 to −0.006), another used air-turbine hand piece (VEAT4) with (*p* = 0.005, α = 0.05), CI (0.050 to 0.397). The mean vibration transmissions of the new micro motor hand piece (VEMM4) was statistically different from new micro motor hand piece (VEMM1) with (*p* = 0.015, α = 0.05), CI (−0.534 to −0.039), another new micro motor hand piece (VEMM2) with (*p* = 0.009, α = 0.05), CI (−1.024 to −0.107), used micro motor hand piece (VEMM3) with (*p* = −0.000, α = 0.05), CI (−0.351 to −0.130), new air-turbine hand piece (VEAT1) with (*p* = 0.001, α = 0.05), CI (−0.787 to −0.168), new air-turbine hand piece (VEAT2) with (*p* = 0.000, α = 0.05), CI (−0.969 to −0.324), used air-turbine hand piece (VEAT3) with (*p* = 0.000, α = 0.05), CI (−0.833 to −0.248). Mainly, the vibration transmissibility of used micro motor hand piece (VEMM4) differed due to obsoleted and old brand.

Another major observation of the study was that there exists a definite difference in vibration transmissibility between new and used hand pieces of both MM and AT. The newly emerged technologies and instruments such as digital impressions [41], laser technologies [42], and other many future advancements in this area have resulted in lessening pain and quick relief, reducing discomfort for both dental surgeons and their patients. The complete analysis is summarized in Table 12.

### 3.4. Discussion of the Results Extracted

The study showed novelty in introducing vibration transmissibility and amplitudes with dental air-turbines and micro-motors. The study showed results that were statistically tested and verified in the light of existing published literature. Firstly, the study examined the statistical testing of mean vibration amplitudes of micro motors and air-turbine hand pieces during a tooth’s drilling by a dental surgeon. An overall comparative analysis of both hand pieces based on their average vibration amplitudes and transmissibility along with distinct grip forces are briefly discussed as below:

- **Result no. 1:** The variance in average vibration amplitudes of twelve hand pieces did not show equality in independent observations of 1/3rd octave band vibration amplitudes with F (11, 252) = 54.017, *p* = 0.000, and α = 0.05. Later, the Welch ANOVA output showed a significant difference in means, and variance between the means are not equal with F (11, 98.842) =5.938, *p* = 0.000 and α = 0.05. A post-hoc comparison Games–Howell test showed that two used hand pieces coded as MUD and AUA show a difference in the average vibration amplitudes.
  **Possible causes of disagreement:** The reason for the difference in micro motor used hand piece (MUD) and used air-turbine hand piece (AUA) is the overuse of both hand pieces. The MUD was used for more than two and a half years. The AUA was used for more than three years, impacting the dental professionals’ overall professional idling efficiencies. This is why the idling efficiency of both used hand pieces were different from the other hand pieces under study.
- **Result no. 2:** The study found all grasping conditions of micro motor and air-turbine at 1/3–octave band vibration amplitude at 22 different frequencies testing homogeneity of variances in two hand pieces, *p*-value < 0.05 depicting equal variances were not assumed. The study has also shown that output is statistically significant in all grasping conditions with the help of an independent t-test on air-turbine and micro-motors. Hence, both devices differ from each other on the pretext of comparison of corresponding grasping style.
  The Levene test showed that computed variances for 1/3rd–octave band vibrations amplitudes for various hand pieces various grasping conditions are not equal with F (7, 168) = 20.922, *p* = 0.000, and α = 0.05. The Welch ANOVA output showed that there is a significant difference in means, and variances between the means are not equal with F (7, 70.486) = 10.788, *p* = 0.000, and α = 0.05. A post-hoc comparison Games–Howell test showed that the tight grasping style in the case of micro motor devices differs from the rest of the styles.
  **Possible causes of disagreement:** These electrically driven micro motors are edging over the air-turbine while grasping and drilling in dental operations. Electric micro motors, when held tightly, give a hand piece strength of 0.18 million rpm. Hence, it is reasonable that this particular grasping style will differ from others.
- **Result no. 3:** This is the main crux of the present investigation. The vibration transmissibility was evaluated on four different micro motors and 4 air-turbine hand pieces with 22 different frequencies. A study of eight hand pieces measuring 1/3rd–octave band vibrations transmissions during drilling with irrigant injection at position two for varying transmission points by the dentist showed a resonance frequency range less than unity. This indicates that most of the vibrations were absorbed through the dental workers’ fingers, palms, and hands. The lower the transmissibility range, the higher is the absorption by the body limbs of dental workers.
  The Levene test showed that computed variances for 1/3rd–octave band vibrations amplitudes for various grasping conditions of both hand pieces are not equal with F (7, 168) = 12.673, *p* = 0.000, and α = 0.05. The Welch ANOVA output showed that there is a significant difference in means, and variances between the means are not equal. F (7, 69.933) = 18.531, *p* = 0.000 and α = 0.05. A post-hoc comparison Games–Howell test showed that vibration transmissibility of used micro motor hand piece (VEMM4) differs from others.
  **Possible causes of disagreement:** The reason for the difference is the obsolete and old hand piece. This micro motor was observed to be old, overused, and noisy during experimentation. The vibrancy of offhand tools must remain declared according to after EC Machinery Directive. The lesson handbook concerning the desktop should contain the weighted RMS acceleration worth according to who the missile is subjected to if it exceeds 2.5 m/s^2^. If the acceleration price does not outdo 2.5 m/s^2^, it must be mentioned.
  As aforesaid, there are many alternative treatments, technologies, and instruments that can provide quick relief, less pain, and less discomfort for dentists and their patients. These include techniques like laser technology, digital impressions, and intraoral scanners. Drafting direct images with titanium dioxide powder-free intraoral scanning, gypsum models etc., are very usable alternatives in this area [41].

## 4. Conclusions

The study found that both vibration amplitudes and vibration transmissibility, when measured at position 2, are on the higher side as compared to position 1. While idling under dental practices, the average resonance frequency in vibrations amplitude for used MMs is more than AT hand pieces. The study has shown that used micro motor and air-turbine shows distinct vibration amplitudes. The reason for the difference in vibration exposure during idling conditions is owing to their obsolescence and over-usage. When drilling is performed on the patients’ dental part, the dentist’s grasp force ranges from mild grasp to moderate grasp to tight grasp. Different grasp forces have a different effect on vibration amplitudes. It increases every time with the tightening of grasping of the hand piece. A post-hoc test Games-Howell test showed that the vibration amplitudes for each grasping style of MM hand piece differ from other grasping styles of AT hand pieces. The resonance frequency range of vibration transmissibility for both used and new hand pieces (for both MMs and ATs) in a majority of the cases is less than unity. Hence, it shows that most dental professionals’ vibrations were absorbed through their fingers, palms, and hands. The lower the transmissibility range, the higher is the absorption by the body limbs of dental workers. Mainly, the vibration transmissibility of a used micro motor hand piece (VEMM4) differs if it is old and obsolete. Another central observation of the study is that there is a definite difference in vibration transmissibility between new and used hand pieces of MM and AT. This study was based only on air-turbine and micro-motors with a limited number of machines on a set of limited patients in the selected clinics. This study can be extended to more patients in some other areas on a similar pattern to correlate the results derived here.

## 5. Policy Implications, Suggestions and Future Scope of the Study

It has been observed that routine exposure to the dentist or dental workers’ vibration has many severe physical, mental, and psychological ill effects. The used hand pieces are more hazardous as compared to newer ones. The study suggests that these hand pieces must be replaced periodically as per the instrument manufacturers’ guidelines and various dental associations like Indian Dental Association (IDA). Another suggestion is that the dentist must be provided with sufficient breaks between operations, especially after every hand piece. The present research work can be further extended by creating control groups offering rest between two operations and another with continuous operations. There is also scope for adding modern techniques like laser vibrometers to make studies exhaustive in the present scenario.

## Figures and Tables

**Table 1:** Number of utilized hand pieces.

S. No.	Type		Rotational Speed (rpm)	Restricted Rotational Speed (rpm)
1	M *	New micro-motor Hand pieces	25,000–40,000	35,000
2	M			
3	A *	New air-turbine Hand pieces	35,000–400,000	
4	A			
5	M	Used micro-motor Hand pieces	25,000–40,000	
6	M			
7	M			
8	M			
9	M			
10	M			
11	M			
12	M			
13	A	Used air-turbine Hand pieces	35,000–400,000	
14	A			
15	A			
16	A			
17	A			
18	A			
19	A			
20	A			

* Note that ‘A’ indicates Air-turbine Hand Piece and ‘M’ indicates Micro Motors.

**Table 2:** Descriptive statistics for 1/3-octave band vibration amplitudes during idling.

Hand Piece		MM						AT					
Usage of Hand Piece		New		Used				New		Used			
Number of hand piece		1	2	3	4	5	6	7	8	9	10	11	12
Code assigned for hand piece		MNA	MNB	MUA	MUB	MUC	MUD	ANA	ANB	AUA	AUB	AUC	AUD
Mean 1/3rd–octave band vibrations amplitudes		0.080	0.059	0.046	0.024	0.030	0.686	0.043	0.024	0.358	0.058	0.032	0.044
Std. Deviation in 1/3rd–octave band vibrations amplitudes		0.070	0.060	0.041	0.034	0.033	0.614	0.053	0.033	0.327	0.071	0.043	0.030
Std. Error in 1/3rd–octave band vibrations amplitudes		0.015	0.013	0.009	0.007	0.007	0.131	0.011	0.007	0.070	0.015	0.009	0.006
95% Confidence Interval for Mean	Lower Bound	0.049	0.033	0.028	0.009	0.015	0.414	0.019	0.009	0.214	0.026	0.013	0.030
	Upper Bound	0.112	0.086	0.065	0.039	0.044	0.958	0.066	0.038	0.503	0.090	0.051	0.057
Minimum vibrations amplitudes		0.000	0.000	0.000	0.000	0.000	0.000	0.000	0.000	0.000	0.000	0.000	0.000
Maximum1/3rd–octave band vibrations amplitudes		0.202	0.185	0.120	0.103	0.119	10.71	0.150	0.091	0.927	0.209	0.127	0.090

**Table 3:** Post hoc analysis for 1/3-octave band vibration amplitudes during idling.

	Hand Piece		MM						AT					
	Usage		New		Used				New		Used			
	Number		1	2	3	4	5	6	7	8	9	10	11	12
Hand piece description	Code		MNA	MNB	MUA	MUB	MUC	MUD	ANA	ANB	AUA	AUB	AUC	AUD
MUD Post hoc Differences	Mean Difference (I–J)		0.606	0.627	0.640	0.662	0.657	-	0.643	0.663	0.328	0.628	0.655	0.642
	Std. Error		0.132	0.131	0.131	0.131	0.131	-	0.131	0.131	0.148	0.132	0.131	0.131
	Sig.		0.006	0.004	0.003	0.002	0.003	-	0.003	0.002	0.553	0.004	0.003	0.003
	95% Confidence Interval	Lower Bound	0.126	0.147	0.161	0.183	0.178	-	0.164	0.184	−0.194	0.148	0.176	0.164
		Upper Bound	1.086	1.106	1.119	1.140	1.135	-	1.123	1.141	0.849	1.108	1.133	1.121
AUA Post hoc Differences	Mean Difference (I–J)		0.278	0.299	0.312	0.334	0.329	−0.328	0.315	0.334	-	0.301	0.327	0.315
	Std. Error		0.071	0.071	0.070	0.070	0.070	0.148	0.071	0.070	-	0.071	0.070	0.070
	Sig.		0.027	0.014	0.009	0.004	0.005	0.553	0.008	0.004	-	0.013	0.005	0.008
	95% Confidence Interval	Lower Bound	0.020	0.042	0.056	0.079	0.073	−0.849	0.059	0.079	-	0.042	0.071	0.059
		Upper Bound	0.536	0.556	0.568	0.589	0.584	0.194	0.572	0.590	-	0.559	0.583	0.570

**Table 4:** 1/3rd-octave band vibration amplitude observations with different grasping conditions.

S.No.	Frequency (Hz)	Mild Grasp		Gentle Grasp		Moderate Grasp		Tight Grasp	
		MM	AT	MM	AT	MM	AT	MM	AT
1	4	0.061102	0.02232	0.045358	0.022622	0.048273	0.022605	0.055188	0.022679
2	5	0.077492	0.014792	0.059547	0.014795	0.058292	0.013569	0.067697	0.02106
3	6.3	0.085361	0.020077	0.075257	0.015571	0.082418	0.016272	0.083462	0.033346
4	8	0.090946	0.02337	0.096737	0.018692	0.108857	0.018911	0.108265	0.039356
5	10	0.096186	0.017983	0.104106	0.019338	0.11251	0.019501	0.12911	0.042234
6	12.5	0.095075	0.014772	0.09945	0.018372	0.105408	0.017963	0.121874	0.035213
7	16	0.064613	0.011493	0.104221	0.012878	0.104044	0.014565	0.108744	0.021135
8	20	0.065636	0.004925	0.085852	0.007521	0.084231	0.009747	0.090106	0.017771
9	25	0.050784	0.003534	0.085755	0.004155	0.074222	0.005725	0.077805	0.013261
10	31.5	0.039617	0.003068	0.069618	0.002796	0.055338	0.003437	0.060045	0.00878
11	40	0.032447	0.002309	0.044357	0.002328	0.046594	0.002319	0.047724	0.004875
12	50	0.031231	0.002249	0.045057	0.002304	0.038382	0.002291	0.051721	0.003068
13	63	0.021891	0.001158	0.035641	0.001158	0.029066	0.001221	0.040975	0.001459
14	80	0.015031	0.000927	0.020398	0.000985	0.019231	0.000927	0.022152	0.0011
15	100	0.017962	0.000812	0.022426	0.000755	0.025223	0.000812	0.023977	0.000812
16	125	0.009328	0.000693	0.011679	0.000693	0.011516	0.000693	0.012676	0.000693
17	160	0.000245	0.000173	0.000173	0.000173	0.000173	0.000173	0.0003	0.000173
18	200	0.000173	0.000173	0.000173	0.000173	0.000173	0.000173	0.000173	0.000173
19	250	0.001625	0	0.001797	0	0.001517	0	0.001625	0
20	315	0.000173	0	0.000173	0	0.000173	0	0.000173	0
21	400	0.000173	0.0001	0.000173	0.000173	0.000173	0.000173	0.000173	0.000173
22	500	0	0	0	0	0	0	0	0

**Table 5:** Descriptive statistics for 1/3-Octave band vibration amplitude with different grasping conditions.

Grasping Conditions	Type of Grip Force		N	Mean Vibration Amplitude	Std. Deviation Vibration Amplitude	Std. Error Mean of Vibration Amplitude
	Type of Hand Piece	Code Assigned to the Hand Piece				
Mild Grasping Position	MM	MMMG	22	0.0390	0.0352	0.0075
	AT	ATMG	22	0.0066	0.0083	0.0018
Gentle Grasping Position	MM	MMGG	22	0.0458	0.0390	0.0083
	AT	ATGG	22	0.0066	0.0080	0.0017
Moderate Grasping Position	MM	MMMoG	22	0.0457	0.0404	0.0086
	AT	ATMoG	22	0.0069	0.0080	0.0017
Tight Grasping Position	MM	MMTG	22	0.0502	0.0436	0.0093
	AT	ATTG	22	0.0122	0.0146	0.0031

**Table 6:** Independent *t*-test for various grasping conditions at two different hand pieces.

Grasping Conditions		Levene’s Test for Equality of Variances		*t*-Test for Equality of Means						
		F	Sig.	t	df	Sig. (2-tailed)	MD *	SED *	95% CID *	
									L *	U *
Mild Grasping Position	Equal variance assumed	45.44	0.000 (Not assumed)	4.202	42.0	0.000	0.032	0.008	0.017	0.048
	Equal variance not assumed			4.202	23.33	0.000 (Ho Not accepted)	0.032	0.008	0.016	0.048
Gentle Grasping Position	Equal variance assumed	42.96	0.000 (Not assumed)	4.620	42.0	0.000	0.039	0.008	0.022	0.056
	Equal variance not assumed			4.620	22.75	0.000 (Ho Not accepted)	0.039	0.008	0.022	0.057
Moderate Grasping Position	Equal variance assumed	39.55	0.000 (Not assumed)	4.424	42.0	0.000	0.039	0.009	0.021	0.057
	Equal variance not assumed			4.424	22.65	0.000 (Ho Not accepted)	0.039	0.009	0.021	0.057
Tight Grasping Position	Equal variance assumed	23.24	0.000 (Not assumed)	3.881	42.0	0.000	0.038	0.010	0.018	0.058
	Equal variance not assumed	-	-	3.881	25.62	0.001 (Ho Not accepted)	0.038	0.010	0.018	0.058

* Where MD, SED, CID, L, and U is the mean difference, standard deviation error difference, confidence interval difference, upper, and lower, respectively.

**Table 7:** Descriptive statistics of 1/3 –Octave Band vibration amplitudes for different grasp forces while drilling with irrigant injection.

Hand Piece		MM				AT			
Usage of Hand Piece		Mild	Gentle	Moderate	Tight	Mild	Gentle	Moderate	Tight
Number of hand Piece		1	2	3	4	5	6	7	8
Code assigned to hand piece		MMMG	MMGG	MMMoG	MMTG	ATMG	ATGG	ATMoG	ATTG
Mean of 1/3rd-octave band vibration amplitude		0.038959	0.045816	0.045719	0.050180	0.006588	0.006613	0.006867	0.012153
Std. Deviation of 1/3rd-octave band vibration amplitude		0.0351685	0.0389952	0.0404009	0.0435919	0.0082956	0.0079690	0.0080147	0.0145510
Std. Error of 1/3rd-octave band vibration amplitude		0.0074979	0.0083138	0.0086135	0.0092938	0.0017686	0.0016990	0.0017087	0.0031023
95% Confidence Interval for Mean	Lower Bound	0.023366	0.028526	0.027806	0.030853	0.002910	0.003080	0.003314	0.005701
	Upper Bound	0.054551	0.063105	0.063632	0.069508	0.010266	0.010146	0.010421	0.018604
Minimum1/3rd-octave band vibration amplitude		0.0000	0.0000	0.0000	0.0000	0.0000	0.0000	0.0000	0.0000
Maximum1/3rd-octave band vibration amplitude		0.0962	0.1042	0.1125	0.1291	0.0234	0.0226	0.0226	0.0422

**Table 8:** Post hoc analysis for various grasping conditions in two different hand pieces.

Hand Piece			MM				AT			
Usage of Hand Piece			Mild	Gentle	Moderate	Tight	Mild	Gentle	Moderate	Tight
Number of Hand Piece			1	2	3	4	5	6	7	8
Code Assigned to Hand Piece			MMMG	MMGG	MMMoG	MMTG	ATMG	ATGG	ATMoG	ATTG
MMMG	Mean Difference (I–J)		-	−0.007	−0.007	−0.011	0.032	0.032	0.032	0.027
	Std. Error		-	0.011	0.011	0.012	0.008	0.008	0.008	0.008
	Sig.		-	0.999	0.999	0.980	0.007	0.007	0.007	0.046
	95% Confidence Interval	Lower Bound	-	−0.043	−0.043	−0.049	0.007	0.007	0.007	0.000
		Upper Bound	-	0.029	0.030	0.027	0.058	0.058	0.058	0.053
MMGG	Mean Difference (I–J)		0.007	-	0.000	−0.004	0.039	0.039	0.039	0.034
	Std. Error		0.011	-	0.012	0.012	0.008	0.008	0.008	0.009
	Sig.		0.999	-	1.000	1.000	0.003	0.003	0.003	0.015
	95% Confidence Interval	Lower Bound	−0.029	-	−0.038	−0.044	0.011	0.011	0.011	0.005
		Upper Bound	0.043	-	0.038	0.035	0.067	0.067	0.067	0.063
MMMoG	Mean Difference (I–J)		0.007	0.000	-	−0.004	0.039	0.039	0.039	0.034
	Std. Error		0.011	0.012	-	0.013	0.009	0.009	0.009	0.009
	Sig.		0.999	1.000	-	1.000	0.004	0.004	0.004	0.021
	95% Confidence Interval	Lower Bound	−0.030	−0.038	-	−0.045	0.010	0.010	0.010	0.003
		Upper Bound	0.043	0.038	-	0.036	0.068	0.068	0.068	0.064
MMGG	Mean Difference (I–J)		0.011	0.004	0.004	-	0.043	0.044	0.044	0.038
	Std. Error		0.012	0.012	0.013	-	0.009	0.009	0.009	0.010
	Sig.		0.980	1.000	1.000	-	0.003	0.003	0.003	0.013
	95% Confidence Interval	Lower Bound	−0.027	−0.035	−0.036	-	0.012	0.012	0.012	0.006
		Upper Bound	0.049	0.044	0.045	-	0.075	0.075	0.075	0.070

**Table 9:** 1/3rd-octave band average vibration transmissions observations.

Hand Piece		MM				AT			
Usage		New		Used		New		Used	
Number		1	2	3	4	5	6	7	8
Code		VEMM1	VEMM2	VEMM3	VEMM4	VEAT1	VEAT2	VEAT3	VEAT4
S. No.	Frequency (Hz)	1	2	5	7	3	4	14	16
1	4	1.332623	0.893539	0.61474	0.205814	0.511174	0.888751	0.61867	0.367738
2	5	0.903326	1.168455	0.454325	0.263473	0.483062	1.149103	0.626443	0.279294
3	6.3	0.780509	1.465982	0.424118	0.282336	0.60051	1.151523	0.707801	0.367199
4	8	0.647043	1.751821	0.401938	0.280732	0.789163	1.264929	0.718729	0.380642
5	10	0.505716	1.841547	0.3298	0.243792	0.939132	1.235758	0.803602	0.321782
6	12.5	0.484758	1.75728	0.33136	0.22856	1.086217	1.232928	1.05872	0.254641
7	16	0.401497	1.551646	0.517135	0.234704	0.851565	1.214412	1.067767	0.204182
8	20	0.431736	1.404339	0.513706	0.223313	0.926529	0.906346	0.862737	0.181025
9	25	0.463981	1.16148	0.520726	0.201863	1.018485	0.876886	1.027158	0.157877
10	31.5	0.494685	0.928678	0.499663	0.274051	1.301875	1.069459	1.107523	0.11347
11	40	0.468912	0.737711	0.44934	0.33411	1.290452	1.132554	1.160653	0.174032
12	50	0.41766	0.582814	0.487399	0.270538	1.259204	1.07415	1.073087	0.215577
13	63	0.416147	0.462888	0.517069	0.218723	0.673837	1.265966	1.072193	0.211517
14	80	0.308406	0.36675	0.451595	0.195745	0.611718	1.00109	1.133893	0.150858
15	100	0.368654	0.295126	0.483951	0.284196	0.507438	0.824739	0.832641	0.121524
16	125	0.29405	0.230249	0.491351	0.091574	0.395968	0.673448	0.768315	0.097135
17	160	1	0.17764	0.495487	0.167836	1	1	1	0.522233
18	200	1	0.139386	0.480472	0.333333	1	1	1	1
19	250	0.333333	0.107947	0.502329	0.408248	0	0	0	0
20	315	0	0.089298	0.484931	0	0	0	0	0
21	400	0	0.068794	0.579865	0	0	0	0	0
22	500	0	0	0	0	0	0	0	0

**Table 10:** Descriptive statistics of 1/3 octave band vibration transmissions of hand pieces.

Hand Piece		MM				AT			
Usage		New		Used		New		Used	
Number		1	2	3	4	5	6	7	8
Code		VEMM1	VEMM2	VEMM3	VEMM4	VEAT1	VEAT2	VEAT3	VEAT4
Mean		0.502411	0.781062	0.455968	0.215588	0.693015	0.861911	0.756361	0.232760
Std. Deviation		0.3353035	0.6354262	0.1213015	0.1083065	0.4244408	0.4431472	0.3998367	0.2207017
Std. Error		0.0714869	0.1354733	0.0258616	0.0230910	0.0904911	0.0944793	0.0852455	0.0470538
95% Confidence Interval for Mean	Lower Bound	0.353746	0.499330	0.402186	0.167568	0.504828	0.665431	0.579083	0.134907
	Upper Bound	0.651076	1.062794	0.509750	0.263609	0.881201	1.058391	0.933638	0.330614
Minimum		0.0000	0.0000	0.0000	0.0000	0.0000	0.0000	0.0000	0.0000
Maximum		1.3326	1.8415	0.6147	0.4082	1.3019	1.2660	1.1607	1.0000

**Table 11:** Post hoc analysis of 1/3 octave band vibration transmission of hand pieces.

Hand Piece			MM				AT			
Usage of Hand Piece			New		Used		New		Used	
Number of Hand Piece			1	2	3	4	5	6	7	8
Code assigned to Hand Piece			VEMM1	VEMM2	VEMM3	VEMM4	VEAT1	VEAT2	VEAT3	VEAT4
VEMMI	Mean Difference (I–J)		-	−0.279	0.046	0.287	−0.191	−0.360	−0.254	0.270
	Std. Error		-	0.153	0.076	0.075	0.115	0.118	0.111	0.086
	Sig.		-	0.612	0.998	0.015	0.716	0.074	0.327	0.058
	95% Confidence Interval	Lower Bound	-	−0.775	−0.203	0.039	−0.559	−0.739	−0.609	−0.005
		Upper Bound	-	0.218	0.296	0.534	0.178	0.020	0.101	0.545
VEMM2	Mean Difference (I–J)		0.279	-	0.325	0.565	0.088	−0.081	0.025	0.548
	Std. Error		0.153	-	0.138	0.137	0.163	0.165	0.160	0.143
	Sig.		0.612	-	0.308	0.009	0.999	1.000	1.000	0.015
	95% Confidence Interval	Lower Bound	−0.218	-	−0.134	0.107	−0.435	−0.611	−0.491	0.077
		Upper Bound	0.775	-	0.785	1.024	0.611	0.449	0.540	1.020
VEMM3	Mean Difference (I–J)		−0.046	−0.325	-	0.240	−0.237	−0.406	−0.300	0.223
	Std. Error		0.076	0.138	-	0.035	0.094	0.098	0.089	0.054
	Sig.		0.998	0.308	-	0.000	0.234	0.007	0.043	0.005
	95% Confidence Interval	Lower Bound	−0.296	−0.785	-	0.130	−0.548	−0.730	−0.595	0.050
		Upper Bound	0.203	0.134	-	0.351	0.074	−0.082	−0.006	0.397
VEMM4	Mean Difference (I–J)		−0.287	−0.565	−0.240	-	−0.477	−0.646	−0.541	−0.017
	Std. Error		0.075	0.137	0.035	-	0.093	0.097	0.088	0.052
	Sig.		0.015	0.009	0.000	-	0.001	0.000	0.000	1.000
	95% Confidence Interval	Lower Bound	−0.534	−1.024	−0.351	-	−0.787	−0.969	−0.833	−0.187
		Upper Bound	−0.039	−0.107	−0.130	-	−0.168	−0.324	−0.248	0.153

**Table 12:** Summarized objectives of the study.

Factor	*p*-Value	Levene Test Ho	Equal Var. Assumed	Testing Method	*p*-Value	Ho	Go for Post-Hoc	Differing Parameter (Age Group)
Vibration exposure using different hand pieces	0.000	Not Accepted	Not Assumed	Welch Test	0.000	Not Accepted	Games–Howell Test	MUD and AUA
different grip positions on the vibration exposure of dentist	0.000	Not Accepted	Assumed	Welch Test	0.000	Not Accepted	Games–Howell Test	Micro Motor with Air-turbine
Vibration Transmissibility	0.000	Not Accepted	Assumed	Welch Test	0.000	Not Accepted	Games–Howell Test	VEMM4 with everyone except VEAT4
